# Interpopulation resource partitioning of Lesser Frigatebirds and the influence of environmental context

**DOI:** 10.1002/ece3.2565

**Published:** 2016-11-10

**Authors:** Rowan Mott, Ashley Herrod, Rohan H. Clarke

**Affiliations:** ^1^ School of Biological SciencesMonash UniversityClaytonVic.Australia

**Keywords:** competition, demography, foraging conditions, intraspecific partitioning, population ecology, prey availability

## Abstract

Conspecific individuals inhabiting nearby breeding colonies are expected to compete strongly for food resources owing to the constraints imposed by shared morphology, physiology, and behavior on foraging strategy. Consequently, colony‐specific foraging patterns that effectively partition the available resources may be displayed. This study aimed to determine whether intraspecific resource partitioning occurs in two nearby colonies of Lesser Frigatebirds (*Fregata ariel*). A combination of stable isotope analysis and GPS tracking was used to assess dietary and spatial partitioning of foraging resources during the 2013 and 2014 breeding seasons. These results were compared to vessel‐derived estimates of prey availability, local primary productivity, and estimates of reproductive output to suggest potential drivers and implications of any observed partitioning. Isotopic data indicated a more neritic source of provisioned resources for near‐fledged chicks at an inshore colony, whereas their offshore counterparts were provisioned with resources with a more pelagic signal. Deep pelagic waters (>200 m) had higher availability of a preferred prey type despite a trend for lower primary productivity. Differences in foraging ecology between the two populations may have contributed to markedly different reproductive outputs. These findings suggest environmental context influences dietary and spatial aspects of foraging ecology. Furthermore, the effect of colony‐specific foraging patterns on population demography warrants further research.

## 1 Introduction

Colonial organisms may gain fitness benefits from their group‐living lifestyle. These benefits include predator defense (Uetz & Hieber, 1994), transmission of social information (Krebs, 1974; Riley, Greggers, Smith, Reynolds, & Menzel, 2005; Robinson, Richardson, Sendova‐Franks, Feinerman, & Franks, 2009), and increased likelihood of finding a mate (Simpson, Smith, & Kelsall, 1987). The ubiquity of coloniality across diverse taxonomic divisions ranging from insects to mammals indicates that benefits may be common and evolutionary successful (Wilkinson, 1988). Yet, coloniality also incurs disadvantages, such as increased parasite burden (Hieber & Uetz, 1990; Møller, 1987) and competition for prey resources (Furness & Birkhead, 1984; Hoogland & Sherman, 1976).

Animals that behave as central place foragers, returning from foraging trips to the same centrally placed nest, roost, or cache, are time‐limited (Wetterer, 1989). There are necessary trade‐offs between time spent commuting to foraging grounds and aspects of the provisioning strategy including load size, time spent foraging, and choice of patch quality (Bakker, Reiffers, Olff, & Gleichman, 2005; Bonser, Wright, Bament, & Chukwu, 1998; Olsson, Brown, & Helf, 2008; Wetterer, 1989). Central place foraging is particularly common for breeding animals, and decisions around these trade‐offs can have important repercussions for dependent offspring (Lewis et al., 2004). Breeding individuals should develop efficient foraging strategies that best suit the needs of their developing young (Boyd, 1999; Hamer, Lynnes, & Hill, 1998; Weimerskirch, Mougey, & Hindermeyer, 1997) while also maintaining their own body condition (Chaurand & Weimerskirch, 1994; McLaughlin & Montgomerie, 1990; Weimerskirch, 1998; Weimerskirch, Cherel, Cuenot‐Chaillet, & Ridoux, 1997) and that of their breeding partner (Tveraa, Sæther, Aanes, & Erikstad, 1998).

Parents that are able to optimize the mass, delivery rate, and nutrient quality of prey delivered to offspring, increase fitness of their young (Lock, Smiseth, & Moore, 2004; Schwagmeyer & Mock, 2008). Not only does provision of adequate food avoid starvation‐mediated mortality, but higher prey delivery correlates with faster growth of the young (Bukaciński, Bukacińska, & Spaans, 1998; Harfenist, 1995), shorter development times (Harfenist, 1995), and higher body mass at independence (Bosch & Vicens, 2006; Harfenist, 1995). Importantly, these qualities confer advantages on the young that may be maintained long after the young have reached independence (Hamer, Furness, & Caldow, 1991; Schwagmeyer & Mock, 2008). Therefore, selection favoring foraging strategies that optimize prey delivery to young is expected to be strong.

Most seabirds breed colonially and behave as central place foragers when breeding because they must return to the colony for incubation or to provision young. Competition from adjacent colonies within the foraging range of a population of seabirds tends to impose limits on total population size (Furness & Birkhead, 1984). This indicates that depletion of prey resources has occurred as a result of metapopulation‐wide foraging activity and that the reduced availability of food for provisioning young sets a limit on the total reproductive output of the metapopulation. Therefore, it might be expected that conspecifics ranging from nearby colonies display divergent patterns in their foraging ecology that would act to minimize overlap in resource use and maximize the rate that they can provision young. Yet, conspecific individuals share similar morphology and physiology, thereby limiting potential for gross differences in foraging ecology. Indeed, some studies find no evidence of resource partitioning among adjacent colonies of conspecific seabirds and other marine organisms (Clarke, Emmerson, & Otahal, 2006; Evans, Dall, Bolton, Owen, & Votier, 2016). Where studies do find evidence for divergent foraging parameters, differences tend to be in spatial aspects of foraging behavior relating to the location of foraging grounds, and the influence of large colonies in governing spatial usage patterns appears greater than for small colonies (Ainley et al., 2004; Grémillet et al., 2004; Thiebot, Cherel, Trathan, & Bost, 2012; Wakefield et al., 2013).

The marine environment is dynamic and conditions that support higher primary productivity or act to aggregate prey may change over temporal scales ranging from minutes to years. Consequently, the foraging strategy of a seabird must be sufficiently flexible so as to subsume environmental change if the individual is to meet its resource requirements and those of its dependant offspring. This may see individuals increase foraging effort when prey is scarce (Ainley, Ford, Brown, Suryan, & Irons, 2003; Burke & Montevecchi, 2009) or switch from a preferred prey species to a more accessible alternative (Hamer et al., 1991; Montevecchi, Benvenuti, Garthe, Davoren, & Fifield, 2009). Similarly, an individual may alter the location where foraging takes place as a result of changes in environmental conditions (Kowalczyk, Reina, Preston, & Chiaradia, 2015; Pinaud, Cherel, & Weimerskirch, 2005).

Using two large populations of Lesser Frigatebirds (*Fregata ariel* Gray) inhabiting nearby islands, it was predicted that interpopulation differences in foraging location would occur. Stable isotope analysis (SIA) and tracking data were used to assess spatial and dietary attributes of foraging. Findings were compared to at‐sea abundance estimates of their primary prey type, local primary productivity, and estimates of reproductive success to infer putative effects of any observed resource partitioning on populations.

## 2 Methods

### 2.1 Study sites

**Figure 1:**
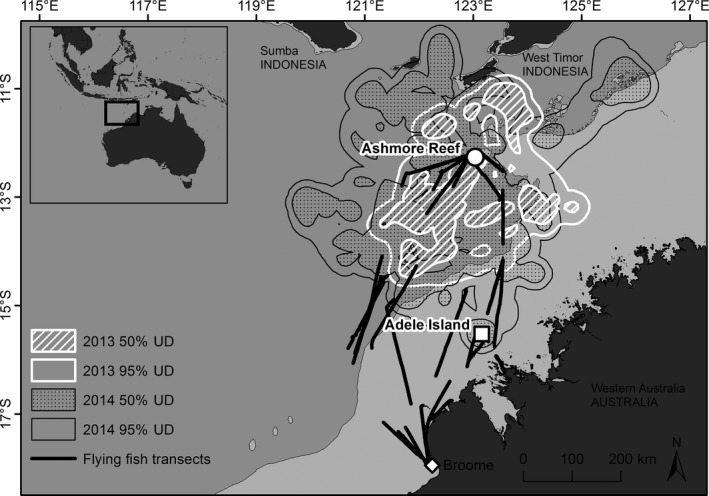
Study region showing the location of the Ashmore Reef and Adele Island study sites. Dark gray shading represents land, whereas mild gray shading represents waters >200 m in depth, and pale gray shading represents shallow waters <200 m in depth. Tracking data of Lesser Frigatebirds from the Ashmore Reef colony are displayed as 50 and 95% utilization distributions for each of the years 2013 and 2014. Kernels were produced following Lascelles et al. (2016) for individual foraging trips and merged to indicate important areas at the colony‐wide scale. The location of flying fish availability transects is also shown. Inset shows the study region in relation to Australia and parts of South‐East Asia

Field work was conducted at two locations in the Browse Basin, northwestern Australia (Figure 1). Ashmore Reef (12° 16′ S 123° 2′ E) is an offshore feature surrounded by pelagic waters >200 m in depth. Adele Island (15° 31′ S, 123° 9′ E) is an inshore island situated in neritic waters <200 m deep. The two locations are ~360 km apart—a distance less than the maximum foraging range of a breeding Lesser Frigatebird (this study)—with no other Lesser Frigatebird colonies between them. At both locations, large colonies of Lesser Frigatebirds trigger designation as BirdLife International Important Bird Areas (IBAs) (Lavers, Miller, Carter, Swann, & Clarke, 2014). Breeding at these sites is highly synchronous with laying occurring from mid‐February through April (Clarke, Carter, Swann, & Thomson, 2011; Clarke, Swann, Mott, Carter, & Herrod, 2013). Thus, timing of courtship, incubation, and chick guarding overlap at both locations. In addition to Lesser Frigatebirds, Ashmore Reef and Adele Island support an additional 22 and 24 species of seabirds and coastal waterbirds, respectively (Clarke & Herrod, 2014; Clarke et al., 2011, 2013; Coate, 1997). At both locations, breeding of Lesser Frigatebirds overlaps temporally with potential food competitors including Great Frigatebirds (*F. minor* Gmelin), Masked (*Sula dactylatra* Lesson), Brown (*S. leucogaster* Boddaert), and Red‐footed Boobies (*S. sula* Linnaeus), as well as many smaller species such as Sooty Terns (*Onychoprion fuscatus* Linnaeus), Greater Crested Terns (*Thalasseus bergii* Lichtenstein), and Brown Noddies (*Anous stolidus* Linnaeus) (Clarke et al., 2011; Coate, 1997). Seabird colonies at each location benefit from protected area designation (Clarke et al., 2011). Ashmore Reef seabird colonies are within Ashmore Reef Commonwealth Marine Reserve, a reserve with an IUCN Ia (strict nature reserve) status and off‐limits to people (Director of National Parks 2013). Adele Island is a Class A Nature Reserve, and human visitation is rare (Coate, 1997). These protective measures extend only to the reef extent. However, interactions with fisheries beyond reserve boundaries are likely to be negligible as very few Western Australian fisheries vessels operate in the region and only those Indonesian vessels that employ traditional fishing methods are permitted in the waters surrounding Ashmore Reef under a Memorandum of Understanding between Australian and Indonesian Governments (Commonwealth of Australia 2002, Fletcher & Santoro, 2015).

### 2.2 Bird capture and processing

Juvenile birds were sampled at Ashmore Reef and Adele Island just prior to fledging from their nests during October and November of 2013 and 2014. Individuals were weighed and fitted with an Australian Bird and Bat Banding Scheme‐supplied metal leg band. From each individual, a sample of five breast feathers was obtained. Feather samples were placed in paper envelopes and stored under dry, dark conditions until analysis. These feathers had been grown while the individual was on the nest and were thus synthesized using locally acquired resources provisioned by both parents. No record of sex was made as it is not possible to distinguish the sex of juvenile Lesser Frigatebirds in the field (James, 2004). Any prey remains spontaneously regurgitated during handling were archived individually and stored frozen (−20°C) following return to the vessel.

Adult Lesser Frigatebirds were captured during the incubation and early chick‐rearing periods. These individuals were captured in March and April of 2013 and 2014 at Ashmore Reef only. Captures were made at night and were facilitated with a bright spotlight to temporarily dazzle an individual attending its nest. Weighing, leg banding, and feather sampling occurred as for juvenile birds. This was followed by the fitting of a global positioning system (GPS) device with Tesa^®^ tape to three central rectrices of the tail (CatTrack 1, Catnip Technologies, Hong Kong: *n* = 54) or by a Teflon leg‐loop harness (HARIER‐4L, Ecotone Telemetry, Sopot, Poland: *n* = 9) (Mott, Herrod, Hodgson, & Clarke, 2015). CatTrack devices were archival‐type loggers sealed in waterproof heatshrink tubing. HARIER‐4L devices had a solar panel and were capable of transmitting stored data via UHF frequencies to a base station that was established on the island, thus negating the need to recapture a bird to recover data. The total weight of the bird‐borne devices was ca. 26 g for CatTrack devices and ca. 15 g for Harier‐4L devices, equivalent to ca. 3.0% (range: 2.6%–3.9%) and ca. 1.8% (range: 1.6%–1.9%) of the bird's body weight, respectively. Finally, a blood sample (~0.5 ml) was collected via brachial venipuncture with a 23‐gauge needle and syringe. This was immediately transferred to a clean 1.5‐ml microtube, and a portable centrifuge was used to separate the sample into its plasma and red blood cell (RBC) components. The plasma portion was transferred to a second clean microtube by pipette, and ethanol was added to both tubes. Whereas some studies indicate storage of samples in ethanol does not alter tissue isotopic ratios (Hobson, Gloutney, & Gibbs, 1997), recent evidence indicates systematic ^13^C enrichment for seabird samples preserved in ethanol (Bugoni, McGill, & Furness, 2008). As such, no comparisons were made between samples stored in ethanol and those stored ethanol‐free. Microtubes containing plasma and RBC were refrigerated upon return to the vessel the following morning and then kept at −20°C once in the laboratory. Any regurgitated prey remains were collected as for juveniles. Individuals fitted with CatTrack devices were recaptured to recover data after a period of 4–25 days.

### 2.3 Stable isotope analysis

Stable isotope analysis (SIA) using ratios of the stable isotopes of nitrogen (^15^N/^14^N) and carbon (^13^C/^12^C) is a method commonly used in research on seabird foraging ecology to detect dietary change in relation to trophic position and/or the location of foraging (Inger & Bearhop, 2008). This approach relies on the predictable manner with which these two isotopic tracers are modified between the proteins of prey and predator. Due to differential elimination between isotopes of nitrogen (Peterson & Fry, 1987), the relative proportion of heavy ^15^N increases with each successive step up the food chain. By contrast, the ratio of different carbon isotopes varies little between trophic levels. Instead, this tracer is used to indicate sources of primary production in the trophic web which are influenced by foraging location (Kelly, 2000). In marine systems, δ ^13^C gradients exist between inshore and offshore locations (Hobson, Piatt, & Pitocchelli, 1994), benthic and pelagic prey sources (Hobson, Ambrose, & Renaud, 1995), and along latitudinal clines (Cherel & Hobson, 2007).

Feather samples were cut into small fragments using scissors to create a homogeneous sample for each individual. A subsample of fragments was rinsed in a 2:1 chloroform:methanol bath followed by two further rinses in methanol. The rinsed product was air‐dried for >48 hr before further trimming with a pizza cutting wheel until the sample resembled a coarse powder. This coarse powder was then weighed (0.7–0.9 mg) into tin capsules for analysis. Ethanol was evaporated off plasma, and RBC samples obtained from adult birds before the samples were freeze‐dried, ground, and weighed as for feather samples. Owing to the small volume of plasma obtained in some instances, lipid extraction was not performed. The mathematical normalization equation presented by Post et al. (2007) for aquatic organisms and followed by Cherel, Connan, Jaeger, and Richard (2014) was used to correct for the presence of lipids in plasma samples. This approach uses the equation δ ^13^C_normalized_ = δ ^13^C − 3.32 + (0.99 × C:N) to correct the measured δ ^13^C value for the presence of lipids based on the recorded ratio of the mass of carbon to nitrogen (C:N) in the sample. No correction was undertaken for feather and RBC samples as C:N was consistently low (<3.5) indicating low lipid content (Post et al., 2007).

An ANCA‐GSL2 elemental analyzer was used for sample analysis, and a Hydra 20:22 isotope‐ratio mass spectrometer (Sercon Ltd., Cheshire, UK) analyzed the resultant CO_2_ and N_2_ gases. A laboratory standard separated every five unknown samples. The equation δ ^13^C or δ ^15^N = (*R*_sample_/*R*_standard_) − 1, where *R* = the ratio of the heavy isotope to the light isotope (^13^C/^12^C or ^15^N/^14^N) in the sample and standards, was used to derive stable isotope abundances with values expressed in per mille units (‰). The international standards Vienna Peedee Belemnite and atmospheric N_2_ were used for carbon and nitrogen isotopic ratios, respectively.

### 2.4 Identification of regurgitated prey remains

Prey remains were identified to be the lowest taxonomic unit possible. Visual identification was aided using species identification sheets (Food and Agriculture Organization of the United Nations 1974), Allen, Swainston, and Ruse (2009), the online resource Fishbase (www.fishbase.org), and a photographic reference collection of known‐identity prey remains. A key was used to identify flying fish remains to species level (Food and Agriculture Organization of the United Nations 1999). Fish otoliths and hard parts of cephalopods were also recovered from regurgitate samples. Otoliths were used for identification by comparison with a reference collection extracted from known‐identity fish as well as comparison with material in Furlani, Gales, and Pemberton (2007) and Fishbase.

Cephalopod beaks were identified where possible using Lu and Ickeringill (2002) and identification was supported by comparison with images in Chen et al. (2012), Nateewathana (1992), Wolff (1982, 1984), Xavier and Cherel (2009). Cuttlebones were identified by comparison with images in Norman and Reid (2000).

It was not possible to identify all ingested items to species level because some items were partially digested. Consequently, all results are grouped at the family level.

### 2.5 Flying fish availability data

During vessel transit from the port of departure at Broome, Western Australia, to the study sites, the location and number of flying fish flushed from the water surface into flight by the vessel were recorded to a handheld PDA with inbuilt GPS (Nautiz X7; Handheld Group, Lidköping, Sweden). A minimum of two observers were stationed on the bow at all times during daylight hours and recorded all flying fish sighted. Transect strip width was considered unimportant as flying fish are not flushed into flight by vessels at distances beyond which they can be detected. Data were available for two voyages in 2013 (April and November) and three voyages during 2014 (March, April, and November) with approximately 6 days of survey effort per voyage (Figure 1).

Frigatebirds are unable to alight on the water surface (Weimerskirch, Chastel, Barbraud, & Tostain, 2003; Weimerskirch, Le Corre, Jaquemet, Potier, & Marsac, 2004). Consequently, they use surface dipping and surface snatching while in flight as their foraging method (Spear, Ainley, & Walker, 2007). Flying fish are the predominant prey of frigatebirds (Diamond, 1975; Weimerskirch et al., 2004) and the number of flying fish recorded in this manner per kilometer of transect here serves as a proxy of the availability of their primary prey.

### 2.6 Local primary productivity

A qualitative assessment of local primary productivity was made using mean monthly chlorophyll‐α concentration within the maximum foraging range (this study) of Lesser Frigatebirds. Data from the early breeding period (March, April, and May) for 2013 and 2014 were obtained from NASA's MODIS aqua satellite (http://oceandata.sci.gsfc.nasa.gov/opendap/) at a resolution of 4 km.

### 2.7 Estimating reproductive output

The number of active nests was counted at Ashmore Reef and Adele Island during April of 2013 and 2014. The timing of these censuses was planned to coincide with the peak in breeding activity; most birds had already laid by this time and a small number were brooding small chicks. These counts were made using standard seabird population census techniques (Clarke et al., 2011). Briefly, experienced counters moved around the colony and used tripod‐mounted spotting scopes, binoculars and the un‐aided eye, as appropriate, to count the number of attended nests. Handheld twin bank tally counters were used to aid counting.

These counts were repeated in October‐November of each year to record the number of near‐fledged juveniles present at each colony. These data were used to estimate the proportion of active nests in April of each year that had successfully reached this near‐fledging stage. This is the best available measure of breeding success as these remote locations were not visited between the two survey times.

### 2.8 Statistical analysis

#### 2.8.1 Stable isotope analysis

Multivariate analysis of variance (MANOVA) or nonparametric permutational multivariate analysis of variance (PERMANOVA) were conducted to test for overall differences in δ ^13^C and δ ^15^N between Lesser Frigatebirds from different islands in each year. Where significant differences were found, post hoc Tukey's HSD or Kruskal–Wallis tests were conducted to determine which subject groups were responsible for the difference (e.g., Cherel & Hobson, 2007).

#### 2.8.2 Tracking

Tracking data were obtained from 25 Lesser Frigatebirds (17 females and eight males). Foraging trips from 2013 and 2014 were imported into ESRI ArcMAP 10.0. Any movements made by an individual within the reef platform were excluded from analysis because these movements were likely attributable to thermal soaring on reef‐associated thermals rather than foraging activity. It was not possible to monitor the nest status of individuals fitted with remote download loggers. Therefore, to minimize the likelihood of including birds that had failed in their breeding attempt in the analysis, any trip that lasted longer than 12.2 days (the maximum duration of a foraging trip recorded for a CatTracker deployment that was known to be from a bird still engaged in reproductive duties) was considered to indicate breeding failure and that, and all subsequent trips, were excluded from analysis. This duration is consistent with the maximum duration of foraging trips recorded for the congeneric Great Frigatebird at other locations in the Indian Ocean (Weimerskirch et al., 2004). For each individual foraging trip, the maximum range, path distance, duration, and compass bearing of this distal point in relation to the Ashmore Reef colony were extracted. Interannual differences in foraging effort were assessed by comparing foraging range, path distance, and trip duration in the 2 years using mixed‐effects models with sex and year treated as fixed effects and the individual as a random effect. This was implemented in R version 3.2.1 (R Core Team 2015) with the package “lme4” (Bates, Maechler, Bolker, & Walker, 2015) for model construction and *p*‐Values were obtained using likelihood ratio tests of the full model against a partial model lacking the effect in question. Visual inspection of residual and Q‐Q plots were undertaken to assess heteroscedasticity and deviations from a normal distribution. Where this indicated violation of the underlying assumptions of mixed‐effects models, square root transformation of the response variable was carried out. Likewise, the presence of influential data points was assessed by calculating Cook's D in the package “influence.ME” (Nieuwenhuis, te Grotenhuis, & Pelzer, 2012) and any data points returning a Cook's D value exceeding 4/n were excluded before proceeding with the analysis (Van der Meer, Te Grotenhuis, & Pelzer, 2010).

The R package “circular” (Agostinelli & Lund, 2013) was used to investigate clustering of the bearing of the distal point of each foraging trip. To avoid pseudo‐replication in the analysis of the distal bearing data set, only the first complete foraging trip recorded for each individual was included. A Rayleigh test was undertaken to determine whether the distal bearings of each study group were clustered as opposed to uniformly distributed in all directions. When this indicated that study groups showed a clustering in their distal bearing, the mean bearing was calculated and then between groups comparisons were made using a Watson–Williams test.

## 3 Results

### 3.1 Diet of juvenile Lesser Frigatebirds

**Figure 2:**
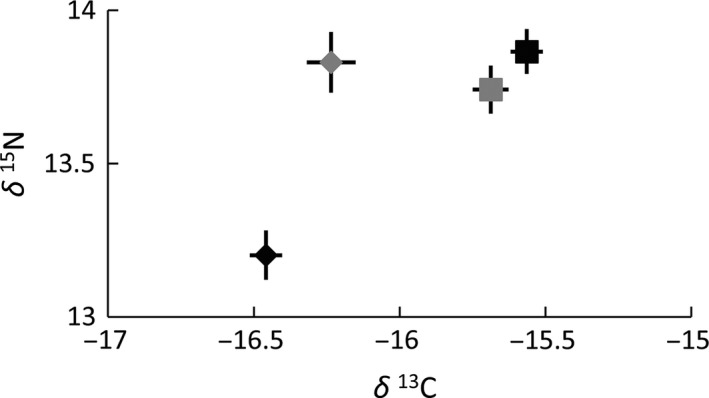
Isotopic bi‐plot depicting the trophic relationships between feather samples from juvenile Lesser Frigatebirds. Samples obtained from Adele Island are represented by square symbols and samples from Ashmore Reef by diamonds. Samples obtained in 2013 are black, whereas those from 2014 are gray. Error bars depict standard error

Overall differences in the isotopic composition of juvenile Lesser Frigatebird feather samples (PERMANOVA: *F*_3,76_ = 24.94, *p *=* *.001) were attributable to samples from Ashmore Reef having a lower mean δ ^13^C value than samples from Adele Island (Figure 2). Feathers obtained from Ashmore Reef in 2013 also had a significantly lower mean δ ^15^N value than mean values for all other juvenile samples (*p *<* *.001 for all three pairwise comparisons) (Figure 2).

At both breeding stations, the remains of fish were present in at least 83% of regurgitate samples from juvenile Lesser Frigatebirds, whereas cephalopod prey occurred in only 27.7% of regurgitate samples at Ashmore Reef and a smaller percentage (26.1%) at Adele Island (Table 1). The fish families Exocoetidae and Hemiramphidae displayed a high frequency of occurrence at Ashmore Reef, each being identified in over a third of regurgitate samples from that colony (Table 1). Hemiramphidae were also frequently identified in samples from Adele Island although the composition of prey remains from that colony showed a more even spread across fish families (Table 1).

**Table 1:** Composition of regurgitated prey remains of juvenile and adult Lesser Frigatebirds expressed as frequency of occurrence (FO %) and numerical abundance (NA %)

Colony	Age			Taxon	*n*	FO %	NA %
Adele Island	Juvenile (*n *=* *61 from 18 samples)	Fish	FO % = 83.3 NA % = 83.6	Carangidae	4	11.1	6.6
				Clupeidae	11	16.7	18.0
				Exocoetidae	2	11.1	3.3
				Hemiramphidae	6	16.7	9.8
				Priacanthidae	0	0.0	0.0
				Scombridae	1	5.6	1.6
				Sillaginidae	0	0.0	0.0
				Unidentified fish	27	55.6	44.3
		Cephalopod	FO % = 27.7 NA % = 16.4	Sepiidae	3	16.7	4.9
				Teuthida	7	22.2	11.5
Ashmore Reef	Juvenile (*n *=* *93 from 23 samples)	Fish	FO % = 95.7 NA % = 89.2	Carangidae	1	4.3	1.1
				Clupeidae	0	0.0	0.0
				Exocoetidae	16	34.8	17.2
				Hemiramphidae	17	39.1	18.3
				Priacanthidae	1	4.3	1.1
				Scombridae	1	4.3	1.1
				Sillaginidae	12	4.3	12.9
				Unidentified fish	35	73.9	37.6
		Cephalopod	FO % = 26.1 NA % = 10.8	Sepiidae	2	8.7	2.2
				Teuthida	8	17.4	8.6
	Adult (*n *=* *29 from 8 samples)	Fish	FO % = 100.0 NA % = 100.0	Exocoetidae	4	50.0	13.8
				Hemiramphidae	4	50.0	13.8
				Scombridae	1	12.5	3.4
				Unidentified fish	20	75.0	69.0
		Cephalopod	FO % = 0.0 NA % = 0.0				
Grand Total					183		

FO % indicates the percentage of individual regurgitate samples from which the prey type was identified. NA % indicates the percentage of the total number of all regurgitated prey items accounted for by that prey type.

### 3.2 Diet and foraging of adult Lesser Frigatebirds

**Figure 3:**
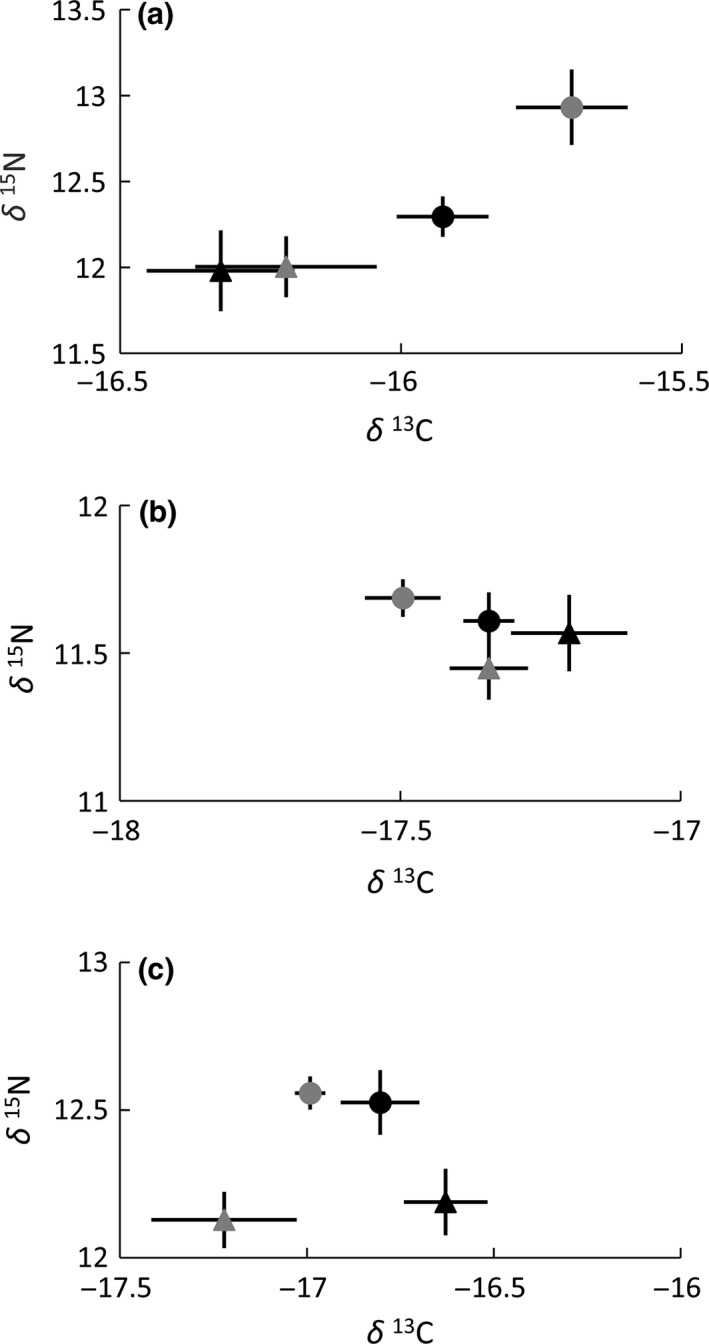
Stable isotope bi‐plots for adult Lesser Frigatebirds captured at Ashmore Reef. Plots depict: (a) feather samples; (b) red blood cells; and (c) normalized plasma. Female samples are represented by circular symbols and male samples by triangles. Samples obtained in 2013 are represented by black symbols and those from 2014 by gray symbols. Plotted values are means with error bars representing the standard error

No significant differences in δ ^13^C or δ ^15^N values between years or sexes were found for feather or RBC samples of adult Lesser Frigatebirds breeding at Ashmore Reef (Feathers PERMANOVA: *F*_3,38_ = 0.23, *p *=* *.74; RBC PERMANOVA: *F*_3,38_ = 0.55, *p *=* *.60) (Figure 3a,b). Plasma samples revealed differences in foraging strategy between years and sexes (MANOVA: *F*_3,35_ = 5.61, *p *<* *.001) (Figure 3c). Interannual variation was evident between male samples with those obtained in 2014 displaying a more pelagic signal typified by depleted ^13^C (*p *<* *.05) (Figure 3c). Between‐sex differences were also found in plasma samples, with males sampled in 2014 foraging on prey of lower trophic level than females in either 2013 or 2014 (*p *<* *.05) (Figure 3c). Similarly, males sampled in 2013 also fed on prey of lower trophic level than females sampled in 2014 (*p *<* *.05) (Figure 3c).

The notable difference between the diet of adult Lesser Frigatebirds compared to juveniles was the apparent lack of cephalopods in adult regurgitate samples (Table 1). Similar to the composition of regurgitate samples of juveniles from Ashmore Reef, Exocoetidae and Hemiramphidae were the most commonly identified family of fish in samples from adult Lesser Frigatebirds (Table 1).

There were no significant differences in the maximum range, path distance, or duration of foraging trips between years (Likelihood ratio test Range: $\chi_{1}^{2}$ = 0.778, *p *=* *.377; Path distance: $\chi_{1}^{2}$ = 3.216, *p *=* *.073; Duration: $\chi_{1}^{2}$ = 3.207, *p *=* *.073) (Table 2).

**Table 2:** Mean attributes of foraging trips undertaken by Lesser Frigatebirds breeding at Ashmore Reef. Values are means (±SD)

Group	Range (km)	Path distance (km)	Duration (days)	Clustered	Mean bearing (°)	Rayleigh test
Females 2013	92.8 (94.7)	820.4 (1112.9)	2.3 (3.4)	No	N.A.	Test statistic = 0.80 *p *=* *.07
Males 2013	166.1 (39.1)	868.4 (249.3)	2.3 (0.7)	No	N.A.	Test statistic = 0.47 *p *=* *.45
Females 2014	104.3 (107.9)	561.8 (827.3)	1.5 (2.4)	Yes	209.1	Test statistic = 0.55 *p *=* *.03
Males 2014	102.8 (96.4)	632.6 (559.6)	1.8 (1.7)	Yes	208.0	Test statistic = 0.87 *p *=* *.04

The mean bearing refers to the direction of the distal point of the first foraging trip recorded for each individual (0° = north, 90° = east, 180° = south, and 270° = west) in relation to the breeding colony. The clustered column indicates whether a Rayleigh test found that the trips were focused along a particular bearing or whether the trips were scattered.

The distribution of distal bearings of trips undertaken by male and female Lesser Frigatebirds was not clustered in 2013, and these groups were excluded from the Watson–Williams test (Table 2). The distal bearing of trips undertaken by males and females in 2014 displayed clustered distributions with trips centered toward the south‐southwest (Table 2). There was no significant difference in the mean bearing between these groups (Watson–Williams test: *F*_1,13_ = 0.001, *p *=* *.975).

### 3.3 Flying fish availability

Mean flying fish availability was higher in pelagic waters (2.904 fish/km) than neritic waters (0.824 fish/km) (*t* = −3.56, *df* = 4, *p *=* *.024). No significant differences in flying fish availability were observed between years (2013: 0.946 fish/km; 2014: 2.710 fish/km; *t* = 2.63, *df* = 3, *p *=* *.079). However, flying fish availability was greater at all sampling points in 2014 than for 2013 excepting availability in pelagic waters in November 2013 (Table 3). Flying fish availability was generally low in April 2013 and remained low in neritic waters in November 2013 (Table 3).

**Table 3:** Flying fish observation data as a function of location (neritic <200 m deep waters and pelagic >200 m waters) and survey period

Sector/period	Flying fish availability (fish/km)	SD among 10 km segments	Number of flying fish	Survey effort (km)
Neritic April 2013	0.288	1.422	161	559.9
Neritic November 2013	0.379	0.674	130	343.3
Neritic March 2014	0.533	0.711	163	533.1
Neritic April 2014	2.159	3.651	710	328.8
Neritic November 2014	1.071	3.022	446	416.3
Pelagic April 2013	0.325	0.547	104	319.5
Pelagic November 2013	3.772	3.871	1017	269.6
Pelagic March 2014	2.560	4.381	1021	398.9
Pelagic April 2014	4.864	9.528	1801	370.3
Pelagic November 2014	2.907	6.516	971	334.0

Flying fish availability is the assumed availability of flying fish to frigatebirds using the number of individual fish recorded as being put to flight in response to the passage of the research vessel per kilometer of survey transect. Standard deviation per kilometer is a measure of how patchily flying fish were distributed at a 10‐km scale. Higher values indicate some areas would be highly profitable foraging locations, whereas low values indicate a more even distribution of flying fish across surveyed area (note only complete 10‐km survey sections were included in the calculation of SD). Number of flying fish is the total number recorded for the research voyage, and survey effort is the total transect length travelled.

Flying fish availability along 10‐km transect segments was significantly more patchy in pelagic waters than in neritic waters (as measured by variation in standard deviation: *t* = −2.81, *df* = 4, *p *=* *.049) (Table 3). Although this measure tended to be higher in 2014 than 2013, the interannual difference was not statistically significant (*t* = −2.46, *df* = 3, *p *=* *.091).

### 3.4 Local primary productivity

Mean chlorophyll‐α concentration within the maximum foraging range of Lesser Frigatebirds from Ashmore Reef during the early breeding periods was consistent between years (2013 = 0.18 ± 0.02 mg/m^3^ and 2014 = 0.18 ± 0.01 mg/m^3^). At inshore Adele Island, mean chlorophyll‐α concentrations of 0.3 ± 0.02 mg/m^3^ and 0.35 ± 0.04 mg/m^3^ occurred in 2013 and 2014, respectively.

### 3.5 Reproductive output

As a measure of reproductive output, significantly more nests reached the near‐fledging stage at Ashmore Reef than did at Adele Island in both years (2013: $\chi_{1}^{2}$ = 54.27, *df* = 1, *p *<* *.001; 2014: $\chi_{1}^{2}$ = 15.27, *df* = 1, *p *<* *.001) (Table 4). Breeding success was similar at Ashmore Reef in both years ($\chi_{1}^{2}$ = 1.22, *df* = 1, *p *>* *.05) (Table 4). However, breeding success was higher at Adele Island in 2014 when compared with 2013 ($\chi_{1}^{2}$ = 26.41, *df* = 1, *p *<* *.001) (Table 4).

**Table 4:** Estimates of nesting success at Ashmore Reef and Adele Island during 2013 and 2014 based on nesting activity at the beginning and end of the breeding period

Location	Year	Number of attended nests (April)	Number of near‐fledged juveniles (November)	Proportion of successful nests
Adele Island	2013	2,835	100^a^	3.53
	2014	4,254	1,520	35.73
Ashmore Reef	2013	2,371	1,520	64.12
	2014	2,098	1,621	77.26

a Count conducted in October.

## 4 Discussion

Evidence for intraspecific differences in resource utilization between breeding stations was apparent. The stable isotope values of feather samples obtained from near‐fledged juveniles demonstrated clear differences in dietary provisioning during a period when the resource demands of a colony are at their peak (Croxall, Prince, & Ricketts, 1985; Simons & Whittow, 1984) creating conditions favoring competition. For colonies of conspecific individuals ranging from nearby locations, a high degree of competition for prey resources is expected due to the constraints imposed by shared morphology, physiology, and general behaviors. To maximize foraging success, it is common for individuals to display colony‐specific foraging strategies that act to partition available resources, for example, New Zealand Fur Seals *Arctocephalus forsteri* (Baylis, Page, & Goldsworthy, 2008), Cape Gannets *Morus capensis* (Grémillet et al., 2004), Northern Gannets *Morus bassanus* (Wakefield et al., 2013), Cory's Shearwater *Calonectris borealis* (Ceia et al., 2015), Rockhopper Penguins *Eudyptes chrysocome chrysocome* (Masello et al., 2010) and *E. c. filholi* (Thiebot et al., 2012), Magellanic Penguins *Spheniscus magellanicus* (Masello et al., 2010), and Gentoo Penguins *Pygoscelis papua* (Masello et al., 2010) (but see Evans et al. (2016), where substantial overlap was observed from three nearby <4 km colonies of European Shags *Phalacrocorax aristotelis*). In the present study, juveniles raised at the inshore island were provisioned with prey from more neritic locations than their offshore counterparts. This pattern was evident in both years. The offshore site was surrounded by pelagic waters. By contrast, the inshore site was located 131 km from the nearest pelagic waters requiring adults breeding at this site to commute large distances to reach pelagic sectors. Our results suggest that this constrains birds originating at the inshore site to a more inshore foraging pattern whereby they either confine foraging trips to neritic waters or obtain substantial prey resources from neritic waters while commuting to and from the more distant pelagic foraging grounds. Whether this behavior is to avoid competition with conspecific individuals ranging from the offshore colony or is a result of some other factor cannot be ascertained from our data (González‐Solís, Croxall, & Wood, 2000). Despite their relative proximity, it appears that the two colonies display a degree of spatial separation in the location of foraging grounds that is maintained from year to year.

Two families of fish, Exocoetidae (flying fish) and Hemiramphidae (halfbeaks), dominated the diet of juvenile and adult Lesser Frigatebirds from the offshore colony. These families are known to be an important component of the diet of frigatebirds at other locations (Diamond, 1975; Weimerskirch et al., 2004). No single family of fish dominated in terms of frequency of occurrence across regurgitate samples from the inshore colony. This suggests that individuals ranging from this colony may be employing an opportunistic foraging strategy, capturing various prey types when the opportunity arises rather than specializing on a diet of flying fish and their allies (Araújo et al., 2008; MacArthur & Pianka, 1966). The higher availability of flying fish in deep pelagic waters suggests a causative reason as to why flying fish are of lower importance in the diet of individuals from the inshore colony. Switching from a preferred prey type to a more varied diet occurs when the preferred prey is scarce, and this can maintain reproductive success (Suryan, Irons, & Benson, 2000). However, switching from a preferred prey species to a varied diet can also result in a decline in reproductive success indicative of difficulty in capturing sufficient prey resources (Baird, 1990).

Interannual differences in SIA values were detected for the offshore colony only. Feather samples collected during the 2014 breeding season indicated that juveniles were provisioned with higher trophic order prey than juveniles in 2013. Furthermore, plasma samples indicated that during the early breeding season of 2013, adult males foraged on neritic prey resources to a greater extent than in 2014. Together, these findings indicate higher behavioral capacity for foraging flexibility or a greater opportunity or need for flexibility in the offshore population compared to the inshore population (Phillips, Catry, Thompson, Hamer, & Furness, 1997). The tendency for primary productivity to be lower within the foraging range of Lesser Frigatebirds from Ashmore Reef may indicate that flexibility is driven by need rather than other potential factors. Flexibility in foraging strategy can buffer against environmental change leading to higher fitness (Berlincourt & Arnould, 2015; Burke & Montevecchi, 2009).

Although foraging flexibility was evident in SIA data for adult males, there was no indication of between‐year alteration of the foraging range, path distance, or duration of adults tracked during the early breeding period. These parameters are a proxy for foraging effort (Petersen, Ryan, & Grémillet, 2006), and together they indicate that foraging effort was consistent between years. However, the distal bearings of foraging trips during 2013 were not concentrated around a particular bearing as they were in 2014. No area was a favored foraging ground at the colony level, which could indicate that prey resources were uniformly distributed around the colony, or that their distribution was unpredictable (Hamer, Phillips, Hill, Wanless, & Wood, 2001; Weimerskirch, 2007). The magnitude of the difference between availability of flying fish in neritic and pelagic waters during the early breeding period of 2013 was small. This may have made it more profitable for some Lesser Frigatebirds to forage in neritic locations, thereby spreading the burden of intraspecific competition across a wider spatial area with little cost in relation to prey encounter rate while at the same time reducing the need to increase foraging effort. A scattered pattern in the distal bearing could also be responsible for the ^13^C‐enriched values obtained from adult male plasma samples in 2013. If during 2013, instead of concentrating foraging trips over deep pelagic waters to the south‐southwest, Lesser Frigatebirds undertook a higher proportion of trips in more easterly and northerly directions, these trips could take individuals over neritic waters proximate to the Australian, Indonesian, and Timor coastlines. Importantly, these trips over neritic waters could be of the same range, path distance, and duration as trips to pelagic waters, thereby accounting for the similarity in foraging effort in tracking data. Owing to the gradient in δ ^13^C between pelagic and neritic waters (Hobson et al., 1994), these trips would account for the interannual differences observed in isotopic analysis of plasma samples from adult males. Croll et al. (2006) found no change in parental foraging effort of Chinstrap Penguins (*Pygoscelis antarcticus*) from the South Shetland Islands despite year‐to‐year changes in prey availability. This resulted in interannual differences in reproductive output. They suggest that long‐lived seabirds should display a strategy that maximizes lifetime reproductive output rather than increasing foraging effort to ensure the survival of that year's young. In the present study, plasticity in foraging behavior may have enhanced the probability of successfully rearing that year's offspring with little cost to foraging effort. Therefore, no additional costs to future reproductive output were incurred.

Reproductive output was lower at the inshore colony than the offshore colony in both years of the study and this disparity was greatest in 2013 when breeding success was only 3.53% at the inshore colony. No significant interannual difference in breeding success was observed at the offshore colony. The availability of flying fish in the neritic waters surrounding the inshore colony was significantly lower than in pelagic waters and low flying fish availability was a feature particularly apparent during 2013. This was despite a tendency for primary productivity to be higher within the foraging range of Lesser Frigatebirds from the inshore colony. These results could implicate low availability of a preferred prey type as a driver of low reproductive performance at the inshore colony.

Findings of the present study suggest that the two populations have differences in foraging site and prey type that limit competition for prey resources. Furthermore, results indicate that environmental context influences how these differences manifest with differences in prey availability likely responsible for observed differences in diet specificity. Although this study was unable to formally test for the effect of prey availability on reproductive output, the apparent relationship between reproductive output and proximity to foraging grounds with high availability of a preferred prey type warrants further investigation.

## Conflict of Interest

None decalred.

## Data Accessibility

Upon acceptance of the manuscript, data will be archived in the Dryad database. DOIs will subsequently be provided.

## References

[ece32565-bib-0001] Agostinelli, C. , & Lund, U. (2013). R package ‘circular’: Circular Statistics (version 0.4‐7). https://r-forge.r-project.org/projects/circular/

[ece32565-bib-0002] Ainley, D. G. , Ford, R. G. , Brown, E. D. , Suryan, R. M. , & Irons, D. B. (2003). Prey resources, competition, and geographic structure of Kittiwake colonies in Prince William Sound. Ecology, 84(3), 709–723.

[ece32565-bib-0003] Ainley, D. G. , Ribic, C. A. , Ballard, G. , Heath, S. , Gaffney, I. , Karl, B. J. , ··· Webb, S. (2004). Geographic structure of Adélie Penguin populations: Overlap in colony‐specific foraging areas. Ecological Monographs, 74(1), 159–178.

[ece32565-bib-0004] Allen, G. R. , Swainston, R. , & Ruse, J. (2009). Field guide to marine fishes of tropical Australia and south‐east Asia. Perth, Western Australia: Western Australian Museum.

[ece32565-bib-0005] Araújo, M. S. , Guimarães, P. R. , Svanbäck, R. , Pinheiro, A. , Guimarães, P. , dos Reis, S. F. , & Bolnick, D. I. (2008). Network analysis reveals contrasting effects of intraspecific competition on individual vs. population diets. Ecology, 89(7), 1981–1993. 18705384 10.1890/07-0630.1

[ece32565-bib-0006] Baird, P. H. (1990). Influence of abiotic factors and prey distribution on diet and reproductive success of three seabird species in Alaska. Ornis Scandinavica (Scandinavian Journal of Ornithology), 21(3), 224–235.

[ece32565-bib-0007] Bakker, E. S. , Reiffers, R. C. , Olff, H. , & Gleichman, J. M. (2005). Experimental manipulation of predation risk and food quality: Effect on grazing behaviour in a central‐place foraging herbivore. Oecologia, 146(1), 157–167. 16049716 10.1007/s00442-005-0180-7

[ece32565-bib-0008] Bates, D. , Maechler, M. , Bolker, B. , & Walker, S. (2015). Fitting linear mixed‐effects models using lme4. Journal of Statistical Software, 67(1), 1–48.

[ece32565-bib-0009] Baylis, A. M. M. , Page, B. , & Goldsworthy, S. D. (2008). Colony‐specific foraging areas of lactating New Zealand fur seals. Marine Ecology Progress Series, 361, 279–290.

[ece32565-bib-0010] Berlincourt, M. , & Arnould, J. P. Y. (2015). Breeding short‐tailed shearwaters buffer local environmental variability in south‐eastern Australia by foraging in Antarctic waters. Movement Ecology, 3(1), 16. 26236479 10.1186/s40462-015-0044-7 PMC4522076

[ece32565-bib-0011] Bonser, R. O. B. , Wright, P. J. , Bament, S. , & Chukwu, U. O. (1998). Optimal patch use by foraging workers of *Lasius fuliginosus*,* L. niger* and *Myrmica ruginodis* . Ecological Entomology, 23(1), 15–21.

[ece32565-bib-0012] Bosch, J. , & Vicens, N. (2006). Relationship between body size, provisioning rate, longevity and reproductive success in females of the solitary bee *Osmia cornuta* . Behavioral Ecology and Sociobiology, 60(1), 26–33.

[ece32565-bib-0013] Boyd, I. L. (1999). Foraging and provisioning in Antarctic fur seals: Interannual variability in time‐energy budgets. Behavioral Ecology, 10(2), 198–208.

[ece32565-bib-0014] Bugoni, L. , McGill, R. A. R. , & Furness, R. W. (2008). Effects of preservation methods on stable isotope signatures in bird tissues. Rapid Communications in Mass Spectrometry, 22(16), 2457–2462. 18642324 10.1002/rcm.3633

[ece32565-bib-0015] Bukaciński, D. , Bukacińska, M. , & Spaans, A. L. (1998). Experimental evidence for the relationship between food supply, parental effort and chick survival in the Lesser Black‐backed Gull *Larus fuscus* . Ibis, 140(3), 422–430.

[ece32565-bib-0016] Burke, C. M. , & Montevecchi, W. A. (2009). The foraging decisions of a central place foraging seabird in response to fluctuations in local prey conditions. Journal of Zoology, 278(4), 354–361.

[ece32565-bib-0017] Ceia, F. R. , Paiva, V. H. , Ceia, R. S. , Hervías, S. , Garthe, S. , Marques, J. C. , & Ramos, J. A. (2015). Spatial foraging segregation by close neighbours in a wide‐ranging seabird. Oecologia, 177(2), 431–440. 25307415 10.1007/s00442-014-3109-1

[ece32565-bib-0018] Chaurand, T. , & Weimerskirch, H. (1994). The regular alternation of short and long foraging trips in the Blue Petrel *Halobaena caerulea*: A previously undescribed strategy of food provisioning in a pelagic seabird. Journal of Animal Ecology, 63(2), 275–282.

[ece32565-bib-0019] Chen, X. , Lu, H. , Liu, B. , Chen, Y. , Li, S. , & Jin, M. (2012). Species identification of *Ommastrephes bartramii*,* Dosidicus gigas*,* Sthenoteuthis oualaniensis* and *Illex argentinus* (Ommastrephidae) using beak morphological variables. Scientia Marina, 76(3), 473–481.

[ece32565-bib-0020] Cherel, Y. , Connan, M. , Jaeger, A. , & Richard, P. (2014). Seabird year‐round and historical feeding ecology: Blood and feather δ^13^C and δ^15^N values document foraging plasticity of small sympatric petrels. Marine Ecology Progress Series, 505, 267–280.

[ece32565-bib-0021] Cherel, Y. , & Hobson, K. A. (2007). Geographical variation in carbon stable isotope signatures of marine predators: A tool to investigate their foraging areas in the Southern Ocean. Marine Ecology Progress Series, 329, 281–287.

[ece32565-bib-0022] Clarke, R. H. , Carter, M. , Swann, G. , & Thomson, J. (2011). The status of breeding seabirds and herons at Ashmore Reef, off the Kimberley coast, Australia. Journal of the Royal Society of Western Australia, 94, 171–182.

[ece32565-bib-0023] Clarke, J. , Emmerson, L. M. , & Otahal, P. (2006). Environmental conditions and life history constraints determine foraging range in breeding Adélie penguins. Marine Ecology Progress Series, 310, 247–261.

[ece32565-bib-0024] Clarke, R. H. , & Herrod, A. (2014). Seabirds and shorebirds at Ashmore Reef, Cartier Island & Browse Island: Monitoring program for the Montara Well release, ninth post‐impact field survey data summary and progress report. Clayton, Vic.: Monash University.

[ece32565-bib-0025] Clarke, R. H. , Swann, G. , Mott, R. , Carter, M. , & Herrod, A. (2013). Seabirds and shorebirds at Adele Island: Results of targeted counts in April 2013. Melbourne: Monash University.

[ece32565-bib-0026] Coate, K. (1997). Seabird islands: Adele Island, Western Australia. Corella, 21(3), 124–128.

[ece32565-bib-0027] Commonwealth of Australia (2002). Ashmore reef national nature reserve and Cartier Island marine reserve (commonwealth waters) management plans. Canberra, Australia: Environment Australia.

[ece32565-bib-0028] Croll, D. A. , Demer, D. A. , Hewitt, R. P. , Jansen, J. K. , Goebel, M. E. , & Tershy, B. R. (2006). Effects of variability in prey abundance on reproduction and foraging in chinstrap penguins (*Pygoscelis antarctica*). Journal of Zoology, 269(4), 506–513.

[ece32565-bib-0029] Croxall, J. P. , Prince, P. A. , & Ricketts, C. (1985). Relationships between prey life‐cycles and the extent, nature and timing of seal and seabird predation in the Scotia Sea In Siegfried W., Condy P. & Laws R. (Eds.), Antarctic nutrient cycles and food webs (pp. 516–533). Berlin, Heidelberg: Springer.

[ece32565-bib-0030] Diamond, A. (1975). Biology and behaviour of frigatebirds *Fregata* spp. on Aldabra Atoll. Ibis, 117(3), 302–323.

[ece32565-bib-0031] Director of National Parks (2013). North‐west commonwealth marine reserves network management plan 2014‐24. Canberra, Australia: Director of National Parks.

[ece32565-bib-0032] Evans, J. C. , Dall, S. R. X. , Bolton, M. , Owen, E. , & Votier, S. C. (2016). Social foraging European shags: GPS tracking reveals birds from neighbouring colonies have shared foraging grounds. Journal of Ornithology, 157(1), 23–32.

[ece32565-bib-0033] Fletcher, W. J. , & Santoro, K. (2015). Status reports of the fisheries and aquatic resources of Western Australia 2014/15: The state of the fisheries. Perth, WA: Department of Fisheries, Government of Western Australia.

[ece32565-bib-0034] Food and Agriculture Organization of the United Nations (1974). FAO species identification sheets for fishery purposes: Eastern Indian Ocean Fishing Area 57 and Western Central Pacific Fishing Area 71. Rome: Food and Agriculture Organization of the United Nations.

[ece32565-bib-0035] Food and Agriculture Organization of the United Nations (1999). FAO species identification guide for fishery purposes. The living marine resources of the Western Central Pacific. Volume 4. Bony fishes part 2 (Mugilidae to Carangidae). Rome: Food and Agriculture Organization of the United Nations.

[ece32565-bib-0036] Furlani, D. , Gales, R. , & Pemberton, D. (2007). Otoliths of common Australian temperate fish: A photographic guide. Collingwood, Australia: CSIRO Publishing.

[ece32565-bib-0037] Furness, R. W. , & Birkhead, T. R. (1984). Seabird colony distributions suggest competition for food supplies during the breeding season. Nature, 311(5987), 655–656.

[ece32565-bib-0038] González‐Solís, J. , Croxall, J. P. , & Wood, A. G. (2000). Foraging partitioning between giant petrels *Macronectes* spp. and its relationship with breeding population changes at Bird Island, South Georgia. Marine Ecology Progress Series, 204, 279–288.

[ece32565-bib-0039] Grémillet, D. , Dell'Omo, G. , Ryan, P. G. , Peters, G. , Ropert‐Coudert, Y. , & Weeks, S. J. (2004). Offshore diplomacy, or how seabirds mitigate intra‐specific competition: A case study based on GPS tracking of Cape gannets from neighbouring colonies. Marine Ecology Progress Series, 268, 265–279.

[ece32565-bib-0040] Hamer, K. C. , Furness, R. W. , & Caldow, R. W. G. (1991). The effects of changes in food availability on the breeding ecology of great skuas *Catharacta skua* in Shetland. Journal of Zoology, 223(2), 175–188.

[ece32565-bib-0041] Hamer, K. C. , Lynnes, A. S. , & Hill, J. K. (1998). Regulation of chick provisioning rate in Manx Shearwaters: Experimental evidence and implications for nestling obesity. Functional Ecology, 12(4), 625–630.

[ece32565-bib-0042] Hamer, K. C. , Phillips, R. A. , Hill, J. K. , Wanless, S. , & Wood, A. G. (2001). Contrasting foraging strategies of gannets *Morus bassanus* at two North Atlantic colonies: Foraging trip duration and foraging area fidelity. Marine Ecology Progress Series, 224, 283–290.

[ece32565-bib-0043] Harfenist, A. (1995). Effects of growth‐rate variation on fledging of rhinoceros auklets (*Cerorhinca monocerata*). The Auk, 112(1), 60–66.

[ece32565-bib-0044] Hieber, C. S. , & Uetz, G. W. (1990). Colony size and parasitoid load in two species of colonial Metepeira spiders from Mexico (Araneae: Araneidae). Oecologia, 82(2), 145–150. 10.1007/BF00323527 28312657

[ece32565-bib-0045] Hobson, K. A. , Ambrose, W. G. Jr , & Renaud, P. E. (1995). Sources of primary production, benthic‐pelagic coupling, and trophic relationships within the Northeast Water Polynya: Insights from delta^13^C and delta^15^N analysis. Marine Ecology Progress Series, 128, 1–10.

[ece32565-bib-0046] Hobson, K. A. , Gloutney, M. L. , & Gibbs, H. L. (1997). Preservation of blood and tissue samples for stable‐carbon and stable‐nitrogen isotope analysis. Canadian Journal of Zoology, 75(10), 1720–1723.

[ece32565-bib-0047] Hobson, K. A. , Piatt, J. F. , & Pitocchelli, J. (1994). Using stable isotopes to determine seabird trophic relationships. Journal of Animal Ecology, 63(4), 786–798.

[ece32565-bib-0048] Hoogland, J. L. , & Sherman, P. W. (1976). Advantages and disadvantages of Bank Swallow (*Riparia riparia*) coloniality. Ecological Monographs, 46(1), 33–58.

[ece32565-bib-0049] Inger, R. , & Bearhop, S. (2008). Applications of stable isotope analyses to avian ecology. Ibis, 150(3), 447–461.

[ece32565-bib-0050] James, D. J. (2004). Identification of Christmas Island, Great and Lesser Frigatebirds. Birding Asia, 1, 22–38.

[ece32565-bib-0051] Kelly, J. F. (2000). Stable isotopes of carbon and nitrogen in the study of avian and mammalian trophic ecology. Canadian Journal of Zoology, 78(1), 1–27.

[ece32565-bib-0052] Kowalczyk, N. , Reina, R. , Preston, T. , & Chiaradia, A. (2015). Environmental variability drives shifts in the foraging behaviour and reproductive success of an inshore seabird. Oecologia, 178(4), 967–979. 25894092 10.1007/s00442-015-3294-6

[ece32565-bib-0053] Krebs, J. R. (1974). Colonial nesting and social feeding as strategies for exploiting food resources in the Great Blue Heron (*Ardea herodias*). Behaviour, 51(1), 99–134.

[ece32565-bib-0054] Lascelles, B. G. , Taylor, P. R. , Miller, M. G. R. , Dias, M. P. , Oppel, S. , Torres, L. , ··· Small, C. (2016). Applying global criteria to tracking data to define important areas for marine conservation. Diversity and Distributions, 22, 422–431.

[ece32565-bib-0055] Lavers, J. L. , Miller, M. G. R. , Carter, M. J. , Swann, G. , & Clarke, R. H. (2014). Predicting the spatial distribution of a seabird community to identify priority conservation areas in the Timor Sea. Conservation Biology, 28(6), 1699–1709. 24976050 10.1111/cobi.12324

[ece32565-bib-0056] Lewis, S. , Hamer, K. , Money, L. , Griffiths, R. , Wanless, S. , & Sherratt, T. (2004). Brood neglect and contingent foraging behavior in a pelagic seabird. Behavioral Ecology and Sociobiology, 56(1), 81–88.

[ece32565-bib-0057] Lock, J. E. , Smiseth, P. T. , & Moore, A. J. (2004). Selection, inheritance, and the evolution of parent‐offspring interactions. The American Naturalist, 164(1), 13–24. 10.1086/421444 15266367

[ece32565-bib-0058] Lu, C. C. , & Ickeringill, R. (2002). Cephalopod beak identification and biomass estimation techniques: Tools for dietary studies of southern Australian finfishes. Museum Victoria Science Reports. Melbourne, Vic.: Museum Victoria.

[ece32565-bib-0059] MacArthur, R. H. , & Pianka, E. R. (1966). On optimal use of a patchy environment. American Naturalist, 100(916), 603–609.

[ece32565-bib-0060] Masello, J. F. , Mundry, R. , Poisbleau, M. , Demongin, L. , Voigt, C. C. , Wikelski, M. , & Quillfeldt, P. (2010). Diving seabirds share foraging space and time within and among species. Ecosphere, 1(6), 1–28.

[ece32565-bib-0061] McLaughlin, R. L. , & Montgomerie, R. D. (1990). Flight speeds of parent birds feeding nestlings: Maximization of foraging efficiency or food delivery rate? Canadian Journal of Zoology, 68(11), 2269–2274.

[ece32565-bib-0062] Møller, A. P. (1987). Advantages and disadvantages of coloniality in the swallow, *Hirundo rustica* . Animal Behaviour, 35(3), 819–832.

[ece32565-bib-0063] Montevecchi, W. A. , Benvenuti, S. , Garthe, S. , Davoren, G. K. , & Fifield, D. (2009). Flexible foraging tactics by a large opportunistic seabird preying on forage‐ and large pelagic fishes. Marine Ecology Progress Series, 385, 295–306.

[ece32565-bib-0064] Mott, R. , Herrod, A. , Hodgson, J. C. , & Clarke, R. H. (2015). An evaluation of the use of predicted harness spans for correctly fitting leg loop harnesses in seabird research. Waterbirds, 38(4), 420–424.

[ece32565-bib-0065] Nateewathana, A. (1992). Taxonomic studies on loliginid squids (Cephalopoda: Loliginidae) from the Andaman Sea coast of Thailand. Phuket Marine Biological Centre Research Bulletin, 57, 1–40.

[ece32565-bib-0066] Nieuwenhuis, R. , te Grotenhuis, M. , & Pelzer, B. (2012). Influence.ME: Tools for detecting influential data in mixed effects models. The R Journal, 4(2), 38–47.

[ece32565-bib-0067] Norman, M. , & Reid, A. (2000). A guide to squid, cuttlefish and octopuses of Australasia. Collingwood, Vic.: CSIRO Publishing.

[ece32565-bib-0068] Olsson, O. , Brown, J. S. , & Helf, K. L. (2008). A guide to central place effects in foraging. Theoretical Population Biology, 74(1), 22–33. 18550139 10.1016/j.tpb.2008.04.005

[ece32565-bib-0069] Petersen, S. L. , Ryan, P. G. , & Grémillet, D. (2006). Is food availability limiting African Penguins *Spheniscus demersus* at Boulders? A comparison of foraging effort at mainland and island colonies. Ibis, 148, 14–26.

[ece32565-bib-0070] Peterson, B. J. , & Fry, B. (1987). Stable isotopes in ecosystem studies. Annual Review of Ecology and Systematics, 18, 293–320.

[ece32565-bib-0071] Phillips, R. A. , Catry, P. , Thompson, D. R. , Hamer, K. C. , & Furness, R. W. (1997). Inter‐colony variation in diet and reproductive performance of great skuas *Catharacta skua* . Marine Ecology Progress Series, 152, 285–293.

[ece32565-bib-0072] Pinaud, D. , Cherel, Y. , & Weimerskirch, H. (2005). Effect of environmental variability on habitat selection, diet, provisioning behaviour and chick growth in yellow‐nosed albatrosses. Marine Ecology Progress Series, 298, 295–304.

[ece32565-bib-0073] Post, D. M. , Layman, C. A. , Arrington, D. A. , Takimoto, G. , Quattrochi, J. , & Montaña, C. G. (2007). Getting to the fat of the matter: Models, methods and assumptions for dealing with lipids in stable isotope analyses. Oecologia, 152(1), 179–189. 17225157 10.1007/s00442-006-0630-x

[ece32565-bib-0074] R Core Team (2015). R: A language and environment for statistical computing. Vienna, Austria: R Foundation for Statistical Computing.

[ece32565-bib-0075] Riley, J. R. , Greggers, U. , Smith, A. D. , Reynolds, D. R. , & Menzel, R. (2005). The flight paths of honeybees recruited by the waggle dance. Nature, 435(7039), 205–207. 15889092 10.1038/nature03526

[ece32565-bib-0076] Robinson, E. J. H. , Richardson, T. O. , Sendova‐Franks, A. B. , Feinerman, O. , & Franks, N. R. (2009). Radio tagging reveals the roles of corpulence, experience and social information in ant decision making. Behavioral Ecology and Sociobiology, 63(5), 627–636.

[ece32565-bib-0077] Schwagmeyer, P. L. , & Mock, D. W. (2008). Parental provisioning and offspring fitness: Size matters. Animal Behaviour, 75(1), 291–298.

[ece32565-bib-0078] Simons, T. R. , & Whittow, G. C. (1984). Energetics of breeding Dark‐rumped Petrels In Whittow G. C. & Rahn H. (Eds.), Seabird Energetics (pp. 159–181). New York: Plenum Press.

[ece32565-bib-0079] Simpson, K. , Smith, J. N. M. , & Kelsall, J. P. (1987). Correlates and consequences of coloniality in great blue herons. Canadian Journal of Zoology, 65(3), 572–577.

[ece32565-bib-0080] Spear, L. B. , Ainley, D. G. , & Walker, W. A. (2007). Foraging dynamics of seabirds in the eastern tropical Pacific Ocean. Studies in Avian Biology, 35, 1–99.

[ece32565-bib-0081] Suryan, R. M. , Irons, D. B. , & Benson, J. (2000). Prey switching and variable foraging strategies of Black‐legged Kittiwakes and the effect on reproductive success. The Condor, 102(2), 374–384.

[ece32565-bib-0082] Thiebot, J.‐B. , Cherel, Y. , Trathan, P. N. , & Bost, C.‐A. (2012). Coexistence of oceanic predators on wintering areas explained by population‐scale foraging segregation in space or time. Ecology, 93(1), 122–130. 22486093 10.1890/11-0385.1

[ece32565-bib-0083] Tveraa, T. , Sæther, B.‐E. , Aanes, R. , & Erikstad, K. E. (1998). Body mass and parental decisions in the Antarctic petrel *Thalassoica antarctica* : How long should the parents guard the chick? Behavioral Ecology and Sociobiology, 43(2), 73–79.

[ece32565-bib-0084] Uetz, G. W. , & Hieber, C. S. (1994). Group size and predation risk in colonial web‐building spiders: Analysis of attack abatement mechanisms. Behavioral Ecology, 5(3), 326–333.

[ece32565-bib-0085] Van der Meer, T. , Te Grotenhuis, M. , & Pelzer, B. (2010). Influential cases in multilevel modeling: A methodological comment. American Sociological Review, 75(1), 173–178.

[ece32565-bib-0086] Wakefield, E. D. , Bodey, T. W. , Bearhop, S. , Blackburn, J. , Colhoun, K. , Davies, R. , ··· Hamer, K. C. (2013). Space partitioning without territoriality in gannets. Science, 341(6141), 68–70. 23744776 10.1126/science.1236077

[ece32565-bib-0087] Weimerskirch, H. (1998). How can a pelagic seabird provision its chick when relying on a distant food resource? Cyclic attendance at the colony, foraging decision and body condition in Sooty Shearwaters. Journal of Animal Ecology, 67(1), 99–109.

[ece32565-bib-0088] Weimerskirch, H. (2007). Are seabirds foraging for unpredictable resources? Deep Sea Research Part II: Topical Studies in Oceanography, 54(3–4), 211–223.

[ece32565-bib-0089] Weimerskirch, H. , Chastel, O. , Barbraud, C. , & Tostain, O. (2003). Flight performance: Frigatebirds ride high on thermals. Nature, 421(6921), 333–334. 12540890 10.1038/421333a

[ece32565-bib-0090] Weimerskirch, H. , Cherel, Y. , Cuenot‐Chaillet, F. , & Ridoux, V. (1997). Alternative foraging strategies and resource allocation by male and female wandering albatrosses. Ecology, 78(7), 2051–2063.

[ece32565-bib-0091] Weimerskirch, H. , Le Corre, M. , Jaquemet, S. , Potier, M. , & Marsac, F. (2004). Foraging strategy of a top predator in tropical waters: Great frigatebirds in the Mozambique Channel. Marine Ecology Progress Series, 275, 297–308.

[ece32565-bib-0092] Weimerskirch, H. , Mougey, T. , & Hindermeyer, X. (1997). Foraging and provisioning strategies of black‐browed albatrosses in relation to the requirements of the chick: Natural variation and experimental study. Behavioral Ecology, 8(6), 635–643.

[ece32565-bib-0093] Wetterer, J. K. (1989). Central place foraging theory: When load size affects travel time. Theoretical Population Biology, 36(3), 267–280.

[ece32565-bib-0094] Wilkinson, G. S. (1988). Reciprocal altruism in bats and other mammals. Ethology and Sociobiology, 9(2), 85–100.

[ece32565-bib-0095] Wolff, G. A. (1982). A beak key for eight eastern tropical Pacific cephalopod species with relationships between their beak dimensions and size. Fishery Bulletin, 80(2), 357–370.

[ece32565-bib-0096] Wolff, G. A. (1984). Identification and estimation of size from the beaks of 18 species of cephalopods from the Pacific Ocean. NOAA Technical Report NMFS 17: US Department of Commerce, National Oceanic and Atmospheric Administration.

[ece32565-bib-0097] Xavier, J. C. , & Cherel, Y. (2009) Cephalopod beak guide for the Southern Ocean. Cambridge, UK: British Antarctic Survey.

